# Synchronization of accelerated intermittent Theta-Burst-Stimulation (aiTBS) with VNS in difficult-to-treat depression (DTD)

**DOI:** 10.1192/j.eurpsy.2024.1464

**Published:** 2024-08-27

**Authors:** E. Kavakbasi, S. B. Klass, B. T. Baune

**Affiliations:** ^1^Department of Psychiatry, University Hospital Münster, University of Münster, Münster, Germany; ^2^Department of Psychiatry, Melbourne Medical School, The University of Melbourne, Melbourne; ^3^The Florey Institute of Neuroscience and Mental Health, The University of Melbourne, Parkville, Australia

## Abstract

**Introduction:**

Patients with difficult-to-treat depression (DTD) need multimodal treatment with combination of psychotherapy, pharmacotherapy and neuromodulation. In severe cases, combination of neuromodulatory techniques may be considered to achieve symptom relief.

**Objectives:**

To describe a novel treatment approach, which combines VNS in synchronization with accelerated intermittent Theta-Burst-Stimulation (aiTBS) over three weeks in two cases with difficult-to-treat depression.

**Methods:**

In this presentation we describe two cases of DTD, which have been implanted with VNS and did not respond to aiTBS previously. Patients then were offered a synchronized treatment regimen, where each stimulus train of aiTBS was synchronized with ON-time of VNS. To start each train simultaneously with VNS ON-time, we set treatment cycle of each aiTBS and VNS to 19 sec. Patients received 2400-3000 TBS pulses daily for 3 weeks over left dorsolateral prefrontal cortex (DLPFC) at 100% of resting motor threshold.

**Results:**

In the first patient the MADRS score decreased from 37 to 26 (-30%) and in the other patient there was a decrease of MADRS score from 20 to 9 (-55%), which corresponded to remission after 3 weeks of treatment. The synchronized treatment procedure was well-tolerated in both cases. As both patients experienced significant improvement, we planned maintenance treatment in both cases.

**Conclusions:**

Synchronization of aiTBS with VNS is a novel treatment approach in patients with DTD, which can lead to improvement even if patients previously did not respond to aiTBS without synchronization with VNS.

**Disclosure of Interest:**

None Declared

